# 

**Published:** 1867-07

**Authors:** 


### CONTENTS.

ORIGINAL COMMUNICATIONS.

Red Blood-Globules....... 281
A Dental Miscellany....... 286
Care of the Teeth....... 292
Improvement in Artificial Teeth....... 299
Address of Dr. Geo. L. Field....... 304
Case of Acute Trifacial Neuralgia....... 308

PROCEEDINGS OF SOCIETIES.

The Third Semi Annual Meeting of the Central States Dental Association....... 311

EDITORIAL.

As Funny as Ever....... 322
Ohio State Dental Society....... 325

